# Association of Adjuvant Antiviral Therapy with Risk of Cancer Progression and Deaths in Patients with Hepatitis-B-Virus-Related Hepatocellular Carcinoma following Curative Treatment: A Nationwide Cohort Study

**DOI:** 10.1371/journal.pone.0102051

**Published:** 2014-07-15

**Authors:** Yi-Chun Yeh, Chun-Jen Liu, Raymond Nienchen Kuo, Chiu-Ling Lai, Wen-Yi Shau, Pei-Jer Chen, Mei-Shu Lai

**Affiliations:** 1 Graduate Institute of Epidemiology and Preventive Medicine, College of Public Health, National Taiwan University, Taipei, Taiwan; 2 Center for Comparative Effectiveness Research, National Center of Excellence for Clinical Trial and Research, National Taiwan University Hospital, Taipei, Taiwan; 3 Department of Internal Medicine and Hepatitis Research Center, National Taiwan University Hospital, Taipei, Taiwan; 4 Graduate Institute of Clinical Medicine, College of Medicine, National Taiwan University, Taipei, Taiwan; 5 Institute of Health Policy and Management, College of Public Health, National Taiwan University, Taipei, Taiwan; 6 Department of Health Industry Management, School of Healthcare Management, Kainan University, Taoyuan, Taiwan; 7 Medical Affairs Division, Pfizer Limited, New Taipei, Taiwan; MOE Key Laboratory of Environment and Health, School of Public Health, Tongji Medical College, Huazhong University of Science and Technology, China

## Abstract

**Background:**

Limited information about tumor status and the time at which antiviral therapy was initiated may have influenced effect estimation in previous research. The aim of this study was to investigate the effect of antiviral therapies on HBV-related HCC progression and deaths in patients receiving curative treatment based on clear clinical-pathological cancer status and the association of start time of adjuvant antiviral therapy initiation and outcomes.

**Methodology:**

A nationwide inception cohort study of newly diagnosed HCC patients who suffered from viral hepatitis B and received curative HCC therapy as the first course of treatment were identified from the Taiwan Cancer Registry between January 1, 2004, and December 31, 2009. Matched Cox proportional hazards models based on propensity score matching and incorporated time-varying exposure were used to estimate adjusted hazard ratios and 95% confidence intervals (CIs).

**Findings:**

Among 3,855 HCC patients with HBV, antiviral therapy was administered to 490 (12.7%) following curative treatment. Antiviral-treated patients had a higher percentage of young age, early stage, and smaller tumor size of HCC compared with untreated patients. After propensity score matching, treated patients demonstrated a higher risk of HCC progression (hazard ratio, 1.42; 95%CI, 1.20–1.69) and death from all causes (1.45; 1.15–1.82) than untreated patients. Similar results were also obtained in sub-cohort of patients who were alive with cancer-free status at least one year after receiving curative treatment and the sub-cohort of patients with liver resection. The interval length between initiation of antiviral therapy and first-line curative treatment did not show a significant association with all-cause mortality.

**Conclusions:**

This study found that adjuvant antiviral therapy did not reduce the risk of HCC progression or mortality in HBV-related HCC patients after cancer status adjusting.

## Introduction

Curative therapies such as surgical resection, percutaneous ethanol injection (PEI), and radiofrequency ablation (RFA) are regarded as standard first-line treatments for patients who suffer from hepatocellular carcinoma (HCC) [1]–[3]. Nonetheless, HCC still commonly recurs following these treatments [1]. This recurrence can be attributed to intra-hepatic spread of the original tumor, persistent hepatic fibrosis, and viral hepatitis activity [4], [5]. Given that viral hepatitis is a common cause of recurrence and that antiviral therapy can effectively inhibit the viral and inflammatory effects which trigger hepatocarcinogenesis, adjuvant antiviral treatments may be able to prevent HCC relapse [6].

Interferon (IFN) treatment was the first historical antiviral therapy to be administered to hepatitis B virus (HBV) related HCC patients. Many studies have previously reported the effectiveness of IFN in decreasing HCC recurrence and mortality rates [7]–[9]. Recently, however, several large randomized clinical trials have raised doubts regarding the benefits of IFN treatments [10]–[12]. Furthermore, current standardized care for HBV, which has been expanded to include nucleos(t)ide analogues and pegylated interferon [13], has achieved better outcomes than IFN therapies, such as lower HCC incidence and overall mortality rates [14]–[16]. Despite these findings, the efficacy of new antiviral agents in reducing the recurrence rate among HBV-related HCC patients following curative therapy remains a matter of contention [17]–[21]. Furthermore, while a recent nationwide study reported that use of nucleoside analogues is associated with a protective effect against HCC recurrence among patients who received liver resection in Taiwan, several issues are in need of further clarification before a definite conclusion can be reached [22]. For example, whether clear surgical margins and tumor status influenced the estimation of protective effects in that study is particularly uncertain [23], [24]. Moreover, the effects of starting antiviral therapy at different time-points following curative treatment for HCC have yet to be investigated.

The purpose of this study was to evaluate the effect of adjuvant antiviral therapies on HCC progression and mortality among HBV-related HCC patients following curative treatment, as based on clear surgical margins and clinico-pathological features of HCC.

## Methods

### Data sources

We conducted a nationwide inception cohort study by identifying HCC patients from the Taiwan Cancer Registry Database. The Taiwan Cancer Registry collects information from all newly diagnosed cancer patients treated in hospitals with a capacity greater than 50 beds [25]. All hospitals which are major providers of cancer care have been eligible to report to the Long-Form database of the Taiwan Cancer Registry (LF-TCR) since 2002. LF-TCR data includes demographics, clinico-pathological variables, and information related to the first course of HCC treatment. Approximately 81.5% of patients newly diagnosed with HCC were listed in the LF-TCR between 2005 and 2009 [26].

In addition, we obtained complete treatment and disease records from the claims database of the National Health Insurance (NHI) program. The NHI program was a mandatory single-payer social health insurance system. It covers outpatient and inpatient services provided by both the private and public sectors. More than 99% of the Taiwanese population was insured under this program at the end of 2008 [27].

We also consulted the National Death Registry (NDR) database to obtain information regarding the date and cause of death.

### Study population

We identified patients who were first diagnosed with HCC (ICD-O-3: C220) between January 1, 2004 and December 31, 2009. To meet inclusion criteria for this study, patients also had to have been diagnosed with viral hepatitis B (ICD-9-CM: 070.2, 070.3, V026.1) within 1 year of receiving a liver resection, PEI, or RFA as the first course of HCC treatment. Additionally, patients were excluded if they met any of the following criteria: (1) diagnosed with another primary form of cancer between 2004 and 2009; (2) aged <18 years; (3) suffered from co-infection with HBV and HCV; (4) received combination treatment as first-line cancer therapy; (5) possessed an unclear surgical margin; (6) received antiviral therapy before first-line treatment for HCC; (7) suffered from a tumor that had directly invaded adjacent organs or lymph nodes, or had undergone distant metastasis as defined by the American Joint Cancer Committee on Cancer (AJCC) system, 6th edition [28].

Among all eligible patients, 92.5% whose NHI claims data for the first course of HCC treatment corresponded to LF-TCR records, were selected for the final analysis.

### Ascertainment of exposure

The main drug exposures of interest were the antiviral therapies: lamivudine, entecavir, adefovir, telbivudine, and pegylated interferon. We determined the total duration of antiviral therapy use by calculating the number of days each drug was prescribed. Patients who received at least one prescription for any antiviral medication were categorized as a treated group. To investigate the relationship between the start date of antiviral therapy and the risk of HCC progression, treated patients were further subdivided into 4 groups according to the interval between curative treatment and initiation of adjuvant antiviral therapy: <6 months, 6–12 months, 12–24 months, and >24 months. Index date was defined as the starting date of the first course of HCC treatment.

### Ascertainment of outcomes

We assessed two outcomes. The first was HCC progression, which was followed patients received second-line cancer therapy (i.e. surgery, RFA, PEI, trans-arterial embolization [TAE], trans-arterial chemoembolization [TACE], radiotherapy, chemotherapy, or hormonal therapy) or died from all causes. The second outcome considered the all cause mortality, which were followed patients until to the date of death. All patients were followed up until the initiation of second-line HCC treatment, death, or December 31, 2011.

### Ascertainment of covariates

We included the following variables in our analyses: sex, age, clinical stage, tumor size, and type of first course of HCC treatment which was obtained from the LF-TCR database. In addition, we defined 7 comorbidities, including congestive heart failure, cerebrovascular disease, dementia, chronic pulmonary disease, rheumatic disease, diabetes mellitus, and renal disease, which had to have been diagnosed within 1 year prior to the index date, based on Deyo's Charlson Comorbidity Index described by Quan et al [29]. Furthermore, to evaluate the liver status of patients, the presence of liver diseases, including alcoholic liver disease (ICD-9-CM codes: 571.0–571.3) and cirrhosis of the liver without mention of alcohol (ICD-9-CM codes: 571.5–571.6), were also identified. These conditions had to have been diagnosed within 1 year of the index date. We also considered severe complications of liver disease, including ascites (ICD-9-CM codes: 789.5), hepatic encephalopathy (ICD-9-CM codes: 572.2), esophageal varices (ICD-9-CM codes: 456.0–456.2), and hapatorenal syndrome (ICD-9-CM codes: 572.4) provided they had been diagnosed within 1 year posterior to the index date. Information was obtained from the NHI database and all diagnoses were identified from a single report in inpatient files or not less than 2 reports in outpatient files.

### Statistical analysis

We used propensity score methods to create a cohort of matched patients who shared similar observed characteristics. We calculated propensity scores using logistic regression to estimate the probability of each patient receiving antiviral therapy on the basis of sex, age, clinico-pathological tumor covariates, presence or absence of liver diseases, complication of liver disease, and other co-morbidities. Patients with calipers of less than 0.2 standard deviations of the logit of propensity scores were grouped together. The matched cohorts were an attempt to group each patient who received antiviral therapy with up to 4 patients who did not (a 1∶4 match). The degree of balance in measured covariates was compared using the Mantel-Haenszel test for categorical variables and generalized estimating equations (GEE) regression for continuous variables. We also calculated standardized differences to obtain a balance between the treated and untreated groups, adopting a difference of less than 0.1 as an indicator of good balance in the matched cohort.

Outcomes were compared using a matched Cox proportional hazard model to estimate the adjusted hazard ratio (HR) and 95% confidence intervals (CI). To ensure that immortal person-time bias was not introduced by treated patients who lived until receiving antiviral therapy after the first course of treatment for HCC, antiviral therapy was incorporated as a time-dependent variable. Several sensitivity analyses were conducted to assess the robustness of our findings. To improve the validity of our definition for curative HCC therapy, we used the same approach to examine the subset of patients who were alive with cancer-free status at least one year after the index date, the subset of provided they underwent a liver resection and had a clear surgical margin, and the subset of both criteria. Furthermore, analyses were stratified by variables of sex, age, tumor status, type of HCC treatment, as well as the presence or absence of alcoholic liver disease, cirrhosis and DM to further determine the effectiveness of adjuvant antiviral therapy for this subset of cohorts.All analyses were two-sided, and *p* values smaller than 0.05 were considered statistically significant. Statistical analysis was performed using SAS, version 9.2 (SAS Institute Inc., Cary, NC, USA).

### Ethics statement

To comply with regulations related to the privacy of personal electronic data, the identity of each patient was encrypted and all data was analyzed anonymously. The study protocol was approved by the Data Release Review Board from the Collaboration Center of Health Information Application (CCHIA), Department of Health (DOH), and by the Research Ethics Committee of National Taiwan University Hospital (protocol # 201205016RIC).

## Results

### Baseline characteristics and antiviral therapy

**Figure 1:**
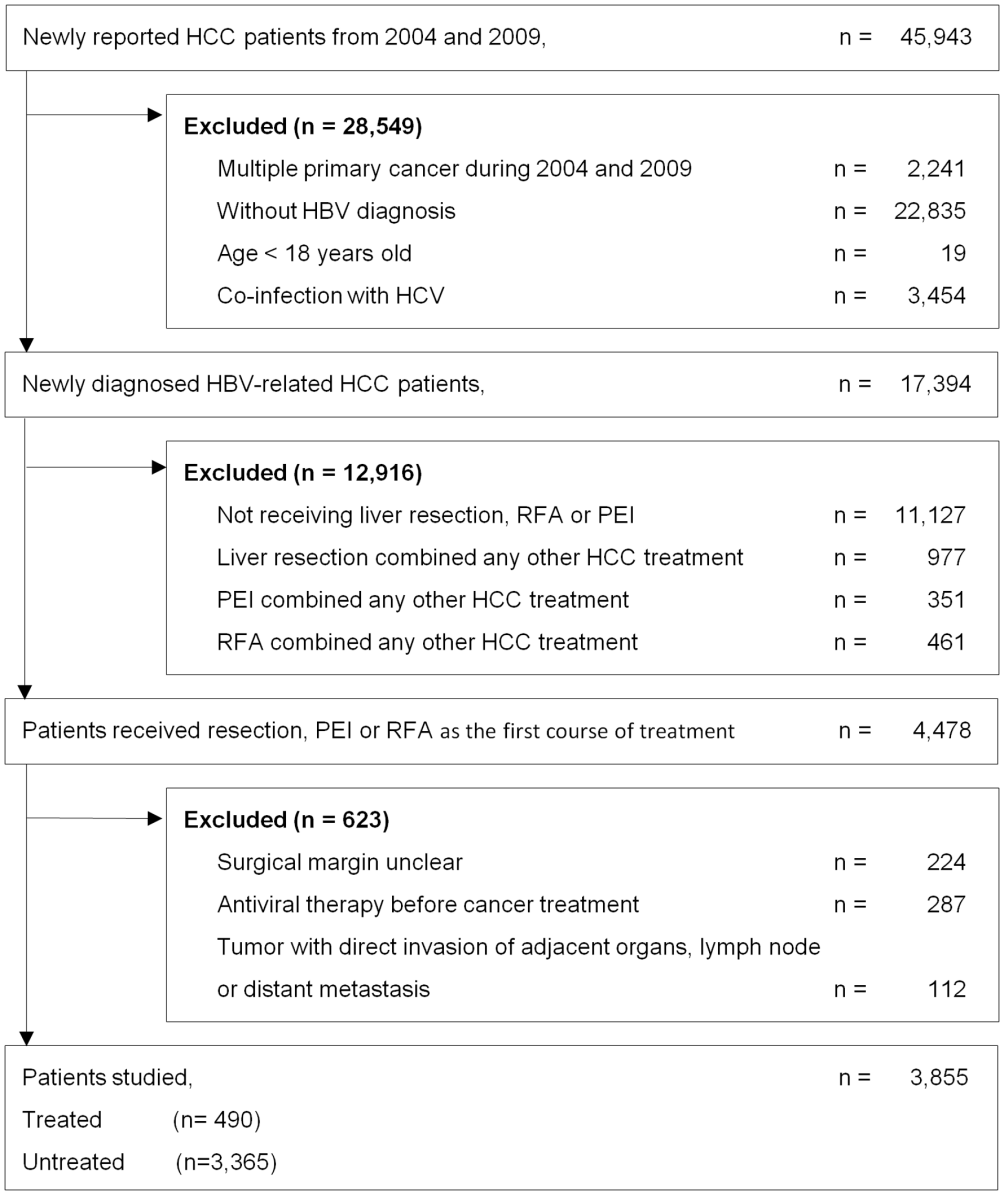
Study flow diagram.

Between January 1, 2004 and December 31, 2009, a total of 45,943 patients with newly diagnosed liver cancer were reported in the LF-TCR. Among them, 3,855 HBV-related HCC patients were selected for analysis. We excluded 2,241 patients who had multiple primary cancer, 19 patients who were less than 18 years old, 26,289 patients who were not diagnosed with HBV or were co-infected with HBV and HCV, 12,916 patients who did not undergo liver resection, RFA or PEI mono-therapy as the first course of HCC treatment; 511 patients who did not meet the criteria of curative therapy and adjuvant antiviral therapy, and 112 patients who suffered from a tumor that had directly invaded adjacent organs or lymph nodes, or had undergone distant metastasis (Figure 1).

In this cohort, 490 (12.7%) patients received adjuvant antiviral therapy. This treatment was initiated at a median of 11.1 months (inter-quartile range [IQR]: 3.9 to 24.6 months) following the first course of HCC therapy. Among treated patients, 280 (57.1%) received entecavir monotherapy, 100 (20.4%) received lamivudine monotherapy, 52 (10.6%) received lamivudine combination therapy, 39 (8.0%) received telbivudine monotherapy, and 19 (3.9%) received other medication. The median duration of antiviral therapy was 10.7 months (IQR: 4.6 to 20.0 months).

Table 1 shows the baseline characteristics of patients before and after propensity score matching. Before propensity score matching, patients who received antiviral therapy were younger, had a higher percentage of clinical stage I or II HCC, possessed a tumor that was smaller than 5 cm, had received RFA treatment, and suffered from cirrhosis. Propensity score matching yielded 490 matched pairs of study patients (490 treated and 1,931 untreated patients). No significant differences were observed between treated and untreated matched groups in terms of baseline characteristics.

**Table 1:** Baseline characteristics of curative HCC patients.

	Before propensity score matching				After propensity score matching			
Follow-up, m	Treated(n = 490)	Untreated(n = 3365)	Standardized Difference, %	p value	Treated(n = 490)	Untreated(n = 1,931)	Standardized Difference, %	p value
Mean(SD)	44.6(21.3)	40.6(23.1)			44.6(21.3)	42.4(22.5)		
Median(IQR)	39.6(29.0–58.7)	37.7(24.3–56.1)			39.6(29.0–58.7)	39.4(26.5–57.3)		
**Interval between HCC therapy and antiviral therapy, m**								
Mean(SD)	16.8(16.4)				16.8(16.4)			
Median(IQR)	11.1(3.9–24.6)				11.1(3.9–24.6)			
**Duration of antiviral therapy, m**								
Mean(SD)	14.6(14.1)				14.6(14.1)			
Median(IQR)	10.7(4.6–20.0)				10.7(4.6–20.0)			
**Gender, No. (%)**								
Male	441(83.9)	2717(80.7)		0.10	411(83.9)	1648(85.3)		0.18
Female	79(16.1)	648(19.3)	8.2		79(16.1)	283(14.7)	4.1	
**Age, mean (SD), y**	55.1(11.6)	55.6(12.5)			55.1(11.6)	55.3(11.2)		
18–49	148(30.2)	1050(31.2)	2.2	0.02	148(30.2)	538(27.9)	5.2	0.12
50–64	241(49.2)	1450(43.1)	12.2		241(49.2)	1005(52.0)	5.7	
65+	101(20.6)	865(25.7)	12.1		101(20.6)	388(20.1)	1.3	
**Clinical stage, No. (%)**								
I	350(71.4)	2211(65.7)	12.4	<0.001	350(71.4)	1390(72.0)	1.2	0.19
II	100(20.4)	636(18.9)	3.8		100(20.4)	405(21.0)	1.4	
III	40(8.2)	518(15.4)	22.6		40(8.2)	136(7.0)	4.2	
**Tumor size, No. (%)**								
< = 2 cm	161(32.9)	736(21.9)	24.8	<0.001	161(32.9)	583(30.2)	5.7	0.47
2–5 cm	243(49.6)	1607(47.8)	3.7		243(49.6)	991(51.3)	3.5	
>5 cm	79(16.1)	975(29.0)	31.1		79(16.1)	328(17.0)	2.3	
unknown	7(1.4)	47(1.4)	0.2		7(1.4)	29(1.5)	0.6	
**Curative treatment, No. (%)**								
Liver resection	392(80.0)	2852(84.8)	12.5	0.001	392(80.0)	1596(82.7)	6.8	0.42
PEI	19(3.9)	159(4.7)	4.2		19(3.9)	66(3.4)	2.5	
RFA	79(16.1)	354(10.5)	16.5		79(16.1)	269(13.9)	6.1	
**Liver disease, No. (%)**								
Alcoholic liver disease	24(4.9)	135(4.0)	4.3	0.36	24(4.9)	82(4.2)	3.1	0.51
Cirrhosis of liver without mention of alcohol	321(65.5)	1896(56.3)	18.9	<.001	321(65.5)	1242(64.3)	2.5	0.56
**Complications of liver disease, No. (%)**								
Ascites	21(4.3)	141(4.2)	0.5	0.92	21(4.3)	68(3.5)	3.9	0.42
Hepatic encephalopathy	15(3.1)	113(3.4)	1.7	0.73	15(3.1)	47(2.4)	3.8	0.41
Esophageal varices	24(4.9)	136(4.0)	4.1	0.37	24(4.9)	74(3.8)	5.2	0.27
Hapatorenal syndrome	<3(<0.6)	13(0.4)	3.4	0.53	<3(<0.6)	<3(<0.2)	4.3	0.29
**Co-morbidity, No. (%)**								
Congestive heart failure	8(1.6)	66(2.0)	2.5	0.62	8(1.6)	35(1.8)	1.4	0.79
Cerebrovascular disease	20(4.1)	143(4.3)	0.8	0.86	20(4.1)	78(4.0)	0.2	0.94
Dementia	3(0.6)	20(0.6)	0.2	0.96	3(0.6)	15(0.8)	2.0	0.69
Chronic pulmonary disease	39(8.0)	262(7.8)	0.6	0.89	39(8.0)	146(7.6)	1.5	0.71
Rheumatic disease	7(1.4)	33(1.0)	4.1	0.36	7(1.4)	28(1.5)	0.2	0.91
Diabetes mellitus	95(19.4)	674(20.0)	1.6	0.74	95(19.4)	368(19.1)	0.8	0.83
Renal disease	15(3.1)	115(3.4)	2.0	0.68	15(3.1)	54(2.8)	1.6	0.74

Abbreviations: PEI, percutaneous ethanol injection; RFA, radiofrequency ablation; SD, standard deviation; IQR, interquartile range.

The exact number of patients below 3 are not specified, in accordance with Taiwan privacy regulations.

### HCC progression and all cause mortality

All patients were followed up for a median of 38.0 months (IQR: 24.3 to 56.1 months), with the exception of the 1,006 (26.1%) patients who died. Table 2 presents the results of propensity score-matched analysis and sensitive analyses. Within matched cohorts, the treated patients had a higher risk of HCC progression (hazard ratio, 1.42; 95%CI, 1.20–1.69) and all-cause mortality (hazard ratio, 1.45; 95%CI, 1.15–1.82) than the untreated group. The risk of HCC progression and all-cause mortality were further compared between entecavir users and lamivudine users. No significant difference was observed between the two subgroups of patients with regard to the risk of HCC progression (entecavir vs. lamivudine∶ hazard ratio, 0.85; 95%CI, 0.50–1.44) and all-cause mortality (entecavir vs. lamivudine∶ hazard ratio, 0.60; 95%CI, 0.32–1.11) (data not shown).

**Table 2:** Hazard ratio for HCC progression and all cause mortality, comparing untreated and treated patients.

Study group/Outcome	N	Event	Person-Years	Incidence rate^a^	Crude Hazard Ratio(95%CI)	Adjusted Hazard Ratio(95%CI)^b^
**Main analysis**						
**All patients**						
**HCC progression**						
Untreated patients	3369	1868	6848.4	272.8	1.00	1.00
Treated patients	490	152	1280.8	118.7	0.45 (0.38 to 0.53)	1.42 (1.20 to 1.69)
**All cause mortality**						
Untreated patients	3369	915	11409.2	80.2	1.00	1.00
Treated patients	490	91	1824.4	49.9	0.63 (0.50 to 0.78)	1.45 (1.15 to 1.82)
**Sensitivity analyses**						
**Patients with cancer-free status at least one year**						
**HCC progression**						
Untreated patients	2270	773	6335.5	122.0	1.00	1.00
Treated patients	431	93	1250.6	74.4	0.60 (0.49 to 0.75)	1.24 (1.00 to 1.55)
**All cause mortality**						
Untreated patients	2270	337	9213.8	36.6	1.00	1.00
Treated patients	431	47	1732.4	27.1	0.75 (0.55 to 1.01)	1.09 (0.80 to 1.50)
**Patients receiving liver resection**						
**HCC progression**						
Untreated patients	2852	1505	6126.2	245.7	1.00	1.00
Treated patients	392	117	1071.9	109.2	0.46 (0.38 to 0.56)	1.46 (1.20 to 1.78)
**All cause mortality**						
Untreated patients	2852	755	9878.4	76.4	1.00	1.00
Treated patients	392	64	1514.6	42.3	0.56 (0.43 to 0.72)	1.37 (1.04 to 1.80)
**Patients receiving liver resection and with cancer-free status at least one year**						
**HCC progression**						
Untreated patients	2004	657	5706.6	115.1	1.00	1.00
Treated patients	349	74	1049.9	70.5	0.60 (0.47 to 0.77)	1.20 (0.94 to 1.54)
**All cause mortality**						
Untreated patients	2004	275	8280.9	33.2	1.00	1.00
Treated patients	349	31	1446.1	21.4	0.65 (0.45 to 0.94)	0.94 (0.64 to 1.37)

a Cumulative incidence rate per 1,000 person years.

b Adjusted Hazard Ratio was based on propensity score matching and incorporated time-dependent exposure.

**Figure 2:**
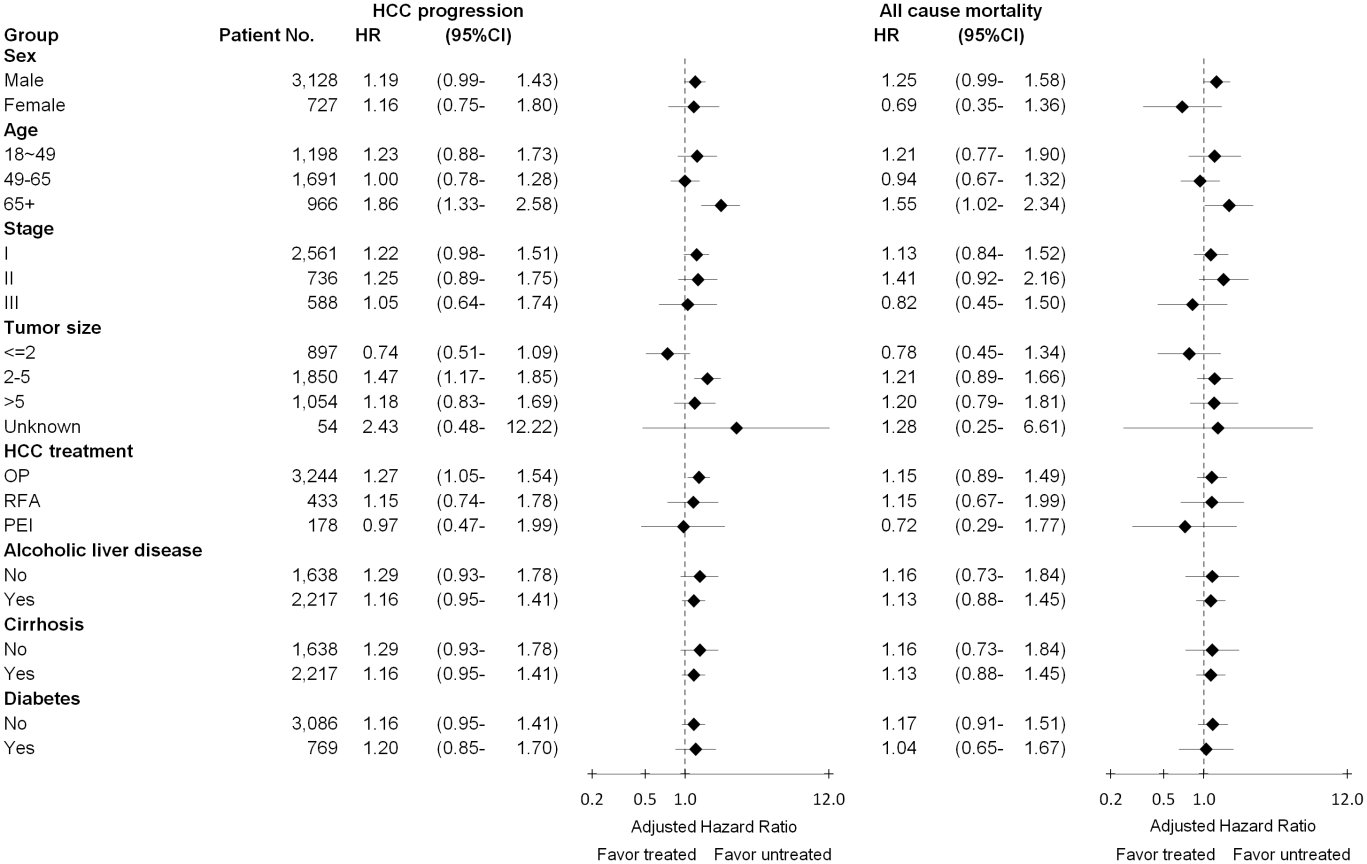
Subgroup analyses comparing treated and untreated on HCC progression and all cause mortality of HBV-related HCC patients by adjusted hazard ratio. Multivariable Cox proportional hazards analyses was adjusted for sex, age, stage, tumor size, curative therapy, alcoholic liver disease, cirrhosis of liver without mention of alcohol, ascites, encephalopathy, esophageal varices, hapatorenal syndrome, congestive heart failure, cerebrovascular disease, dementia, chronic pulmonary disease, rheumatic disease, diabetes mellitus, and renal disease.

Sensitive analyses yielded similar results, the sub-cohort of patients who were alive with cancer-free status at least one year after receiving curative treatment (HCC progression∶ hazard ratio, 1.24; 95%CI, 1.00–1.55; all cause mortality∶ hazard ratio, 1.09; 95%CI, 0.80–1.50), the sub-cohort of patients with liver resection (HCC progression∶ hazard ratio, 1.46; 95%CI, 1.20–1.78; all cause mortality∶ hazard ratio, 1.37; 95%CI, 1.04–1.80) and the sub-cohort of patients who were both with liver resection and alive with cancer-free status at least one year (HCC progression∶ hazard ratio, 1.20; 95%CI, 0.94–1.54; all cause mortality∶ hazard ratio, 0.94; 95%CI, 0.64–1.37). In the stratified analyses, we did not find that adjuvant antiviral therapy was effective at decreasing the risks of cancer progression and all cause mortality, even among patients who differed in terms of sex, age, tumor status, liver disease and DM (Figure 2).

### Relationship between start time of antiviral therapy and HCC progression and all cause mortality

Table 3 shows associations between the start time of antiviral therapy and HCC progression and all cause mortality; however, we found no association between these. For HCC progression, the adjusted HRs was slightly lower among patients who received the first prescription of antiviral therapy within the first six months following HCC treatment (hazard ratio, 0.88, 95%CI: 0.69–1.12).

**Table 3:** Hazard ratio for HCC progression and all cause mortality, comparing untreated patients and patients who received adjuvant antiviral therapy at different time points.

Start time of antiviral therapy from HCC treatment	No of patients	HCC progression		All cause mortality	
		No of event	HR(95%CI)*	No of event	HR(95%CI)*
Untreated	3365	1868	Reference	915	Reference
0 to 6 months	158	69	0.88 (0.69 to 1.12)	44	1.02 (0.75 to 1.39)
6 to 12 months	104	36	1.14 (0.81 to 1.59)	20	0.99 (0.63 to 1.56)
12 to 24 months	100	21	1.29 (0.83 to 1.99)	9	0.68 (0.35 to 1.31)
24+ months	128	26	2.52 (1.68 to 3.79)	18	1.49 (0.93 to 2.41)

*Multivariable Cox proportional hazards analyses was adjusted for sex, age, stage, tumor size, curative therapy, alcoholic liver disease, cirrhosis of liver without mention of alcohol, ascites, encephalopathy, esophageal varices, hapatorenal syndrome, congestive heart failure, cerebrovascular disease, dementia, chronic pulmonary disease, rheumatic disease, diabetes mellitus, and renal disease.

## Discussion

This cohort study evaluated the association between adjuvant antiviral therapy and the rates of HCC progression or all cause mortality of HBV-related HCC patients. Outcomes were determined based on clear surgical margins and clinico-pathological features. This study found that adjuvant antiviral therapy was not effective at decreasing HCC progression and all cause mortality. Furthermore, this study is to our knowledge the first to consider whether the start time of antiviral therapy influences the risk of HCC progression and all cause mortality. The interval length between initiation of antiviral therapy and first-line curative treatment did not show a significant association with all cause survival; however, when antiviral therapy was initiated earlier, adjusted HRs of HCC progression were lower for each treatment group.

The effects of adjuvant antiviral therapies for HBV-related HCC patients have been studied only on a limited basis. The most commonly reported approach has been interferon-based treatment [10]–[12]. With regard to current standardized anti-HBV agents, research investigating nucleoside analogue therapy, including lamivudine or entecavir, has yielded inconclusive results. Three observational studies supported a decrease in overall mortality or HCC recurrence [21], [30], [31]; however, other studies did not [17], [18]. It is possible that the inconsistent results would be due to limited statistical power in those studies. A recent nationwide study in Taiwan found that nucleoside analogues treatments were significantly associated with HCC recurrence for patients receiving liver resection [22]. However, the effects were not found in our study.

In contrast with the previously mentioned study, we certified that HCC patients had been therapeutically cured after undergoing the first-course of HCC treatment by only considering patients who had received curative HCC treatment and adopting additional strict inclusion criteria. Specifically, this study only included patients if they had clear surgical margins and early-stage cancer. Moreover, we added information about clinico-pathological HCC status and found that physicians may consider HCC status in addition to HBV activity [22], when prescribing adjuvant antiviral treatment. For example, this study showed that antiviral-treated patients were with an early stage of HCC, a smaller tumor size, compared with untreated patients among our participants (see Table 1). Certain baseline characteristics are inherently associated with a lower risk of HCC recurrence and mortality. Therefore, certain clinico-pathological factors of HCC would be probably to confound results by biasing the effect estimate towards a protective effect. Nonetheless, after carefully considering what constituted a therapeutic cure and adjusting for clinico-pathological status, this study found that adjuvant antiviral therapies did not decrease the risk of HCC recurrence. The different inclusion criteria and additional information used to adjust for clinico-pathological status might explain the inconsistent results between the previously mentioned study and this paper.

Prior studies which reported the benefits of antiviral therapy found that the average time between initiation of antiviral therapy and first-line HCC treatment was approximately 2 months [20], [21], [30]. However, studies of null association indicated no clear time-point for the initiation of adjuvant antiviral therapy [17]–[19]. Thus, evidence indicating the start time of this treatment influences survival outcomes is limited. Our finding was that when antiviral therapy was initiated earlier, adjusted HRs of HCC progression was lower for each treatment group. The lower risk associated with early use of antiviral therapy might indicate that when these therapies are prescribed within 6 months of first-line HCC treatment, they may be able to prevent the viral and inflammatory effects of viral hepatitis B and in turn prevent HCC recurrence after curative treatment. One possible explanation is that to control the activation of viral hepatitis B and achieve hepatitis B e antigen (HBeAg) seroconversion, some patients required treatment with antiviral therapy for more than 1 year [16]. The incidence of late recurrence increases at 3 years after curative treatment [32]. Thus, for patients receiving early adjuvant antiviral therapy, specifically within 6 months of first-line HCC treatment, the duration of antiviral treatment may be sufficient to achieve antiviral response. As a result, we observed a reduction in the risk of HCC progression. Patients receiving late antiviral therapy may have already been suffering from active viral hepatitis for a long period of time prior to the initiation of anti-HBV therapy. Long-term viral hepatitis activity would probably facilitate HCC recurrence. Furthermore, late antiviral therapy was not in time and had inadequate duration to control the activation of viral hepatitis B. Finally, the negative impact was observed in late antiviral therapy. However, these results were not statistically significant. The association of start time of adjuvant antiviral therapy and HCC progression needs further evaluation and validation.

### Strengths and limitations

This study has several strengths. First, participants were recruited from a nationwide cancer registry and health insurance database. Thus, the study population represents the entire HCC population and real-world clinical practice in Taiwan. Second, to investigate the effectiveness of antiviral therapy in conjunction with different forms of curative HCC treatment, we considered two major curative treatments for HCC, RFA and PEI, in addition to liver resection. Third, to minimize the influence of confounding factors on cancer status and obtain a more accurate definition of second-line HCC treatment for HCC, we used the LF-TCR database and provided detail information on clinico-pathological HCC stauts and complete records of first course cancer treatment. Fourth, we used time-varying exposure to eliminate the risk of immortal time bias and employed propensity score matching to obtain comparable groups of patients.

Nevertheless, this study also has several limitations. First, the LF-TCR and NHI databases do not provide a number of critical clinical measurements, such as biochemical data and liver function. These criteria indicate whether treated patients suffer from more active liver disease and severe biochemical derangement than untreated patients, potentially confounding the benefits of antiviral therapy. For instance, one unmeasured confounding factor could be HBV DNA level. Treated patients may have higher HBV DNA levels, which are expected to be associated with an increase in the risk of HCC recurrence [33]. Thus, we observed the negative impact of antiviral therapy. However, the degree to which unmeasured confounding factors would influence the estimation of effects in these patients would require further investigation. Second, this study was the inability to directly identify HCC progression due to a lack of imaging information. To compensate, we defined HCC progression indirectly by considering the time at which the subsequent course of HCC treatment was initiated or the date of death. In addition, HCC recurrence and mortality within 1 year of curative therapy were regarded as early recurrence caused by the original tumor. Thus, any difference in survival rate detected during this period may be a result of something other than antiviral therapy. To compensate for this problem, we conducted a sensitivity analysis which considered patients who had been alive with cancer-free status at least one year after receiving curative treatment. This analysis did not however effect any change on our results. Finally, although our analyses adjusted for a number of risk factors, other residual confounding issues may have been neglected such as smoking status, drinking or healthy behaviors.

In conclusion, after adjusting for clinical-pathological cancer status which could potentially confound initiation of adjuvant antiviral therapy and study outcomes, this study found that adjuvant antiviral therapy did not confer any benefits related to the reduction of HCC progression and all cause mortality. However, when antiviral therapy was initiated earlier, adjusted HRs of HCC progression were lower for each treatment group. The association of antiviral therapy and HCC progression and deaths on HCC patients and the association of start time of adjuvant antiviral therapy and HCC progression still require further investigation.

## Supporting Information

Checklist S1 **STROBE Statement—Checklist of items that should be included in reports of cohort studies.**
(DOCX)
