# Supplementary material for: Association of Adjuvant Antiviral Therapy with Risk of Cancer Progression and Deaths in Patients with Hepatitis-B-Virus-Related Hepatocellular Carcinoma following Curative Treatment: A Nationwide Cohort Study
Source: PLoS One. 2014 Jul 15;9(7):e102051. doi: 10.1371/journal.pone.0102051 (PMC4098996; doi:10.1371/journal.pone.0102051)
Supplement: Checklist S1 — STROBE Statement—Checklist of items that should be included in reports of cohort studies. (DOCX) [file pone.0102051.s001.docx]

STROBE Statement—Checklist of items that should be included in reports of ***cohort studies***

|  | Item No | Recommendation | Reported in Section |
| --- | --- | --- | --- |
| **Title and abstract** | 1 | (*a*) Indicate the study’s design with a commonly used term in the title or the abstract | Title  Abstract |
|  |  | (*b*) Provide in the abstract an informative and balanced summary of what was done and what was found | Abstract |
| Introduction | | |  |
| Background/rationale | 2 | Explain the scientific background and rationale for the investigation being reported | Introduction |
| Objectives | 3 | State specific objectives, including any prespecified hypotheses | Introduction |
| Methods | | |  |
| Study design | 4 | Present key elements of study design early in the paper | Data sources |
| Setting | 5 | Describe the setting, locations, and relevant dates, including periods of recruitment, exposure, follow-up, and data collection | Data sources  Study population  Ascertainment of Exposure  Ascertainment of outcomes |
| Participants | 6 | (*a*) Give the eligibility criteria, and the sources and methods of selection of participants. Describe methods of follow-up | Data sources  Study population  Ascertainment of Exposure  Ascertainment of outcomes |
|  |  | (*b*) For matched studies, give matching criteria and number of exposed and unexposed | Statistical Analysis |
| Variables | 7 | Clearly define all outcomes, exposures, predictors, potential confounders, and effect modifiers. Give diagnostic criteria, if applicable | Ascertainment of Exposure  Ascertainment of outcomes  Ascertainment of covariates |
| Data sources/ measurement | 8* | For each variable of interest, give sources of data and details of methods of assessment (measurement). Describe comparability of assessment methods if there is more than one group | Data sources  Ascertainment of Exposure  Ascertainment of outcomes  Ascertainment of covariates  Statistical Analysis |
| Bias | 9 | Describe any efforts to address potential sources of bias | Ascertainment of covariates |
| Study size | 10 | Explain how the study size was arrived at | None. This is a nation-wide, population-based register study in Taiwan. |
| Quantitative variables | 11 | Explain how quantitative variables were handled in the analyses. If applicable, describe which groupings were chosen and why | Ascertainment of covariates |
| Statistical methods | 12 | (*a*) Describe all statistical methods, including those used to control for confounding | Statistical Analysis |
|  |  | (*b*) Describe any methods used to examine subgroups and interactions | Statistical Analysis |
|  |  | (*c*) Explain how missing data were addressed | None. Missing data were categorized as missing, for instance, tumor size( see Table 1) |
|  |  | (*d*) If applicable, explain how loss to follow-up was addressed | Ascertainment of outcomes |
|  |  | (*e*) Describe any sensitivity analyses | Statistical Analysis |
| Results | | |  |
| Participants | 13* | (a) Report numbers of individuals at each stage of study—eg numbers potentially eligible, examined for eligibility, confirmed eligible, included in the study, completing follow-up, and analysed | Baseline characteristics and antiviral therapy Figure 1(Study flow diagram) |
|  |  | (b) Give reasons for non-participation at each stage | Baseline characteristics and antiviral therapy Figure 1(Study flow diagram) |
|  |  | (c) Consider use of a flow diagram | Figure 1(*Study flow diagram*) |
| Descriptive data | 14* | (a) Give characteristics of study participants (eg demographic, clinical, social) and information on exposures and potential confounders | Baseline characteristics and antiviral therapy  Table 1 |
|  |  | (b) Indicate number of participants with missing data for each variable of interest | Table 1 |
|  |  | (c) Summarise follow-up time (eg, average and total amount) | HCC progression and all cause mortality  Table 1  Table 2 |
| Outcome data | 15* | Report numbers of outcome events or summary measures over time | HCC progression and all cause mortality  Table 2 |
| Main results | 16 | (*a*) Give unadjusted estimates and, if applicable, confounder-adjusted estimates and their precision (eg, 95% confidence interval). Make clear which confounders were adjusted for and why they were included | Table 2 |
|  |  | (*b*) Report category boundaries when continuous variables were categorized | Table 1 |
|  |  | (*c*) If relevant, consider translating estimates of relative risk into absolute risk for a meaningful time period | None. |
| Other analyses | 17 | Report other analyses done—eg analyses of subgroups and interactions, and sensitivity analyses | HCC progression and all cause mortality  Table2  Figure2 |
| Discussion | | |  |
| Key results | 18 | Summarise key results with reference to study objectives | Discussion |
| Limitations | 19 | Discuss limitations of the study, taking into account sources of potential bias or imprecision. Discuss both direction and magnitude of any potential bias | Strengths and limitations |
| Interpretation | 20 | Give a cautious overall interpretation of results considering objectives, limitations, multiplicity of analyses, results from similar studies, and other relevant evidence | Discussion |
| Generalisability | 21 | Discuss the generalisability (external validity) of the study results | Discussion  This is a nation-wide, population-based register study in Taiwan. Thus, the study population represents the entire HCC population and real-world clinical practice in Taiwan. |
| Other information | | |  |
| Funding | 22 | Give the source of funding and the role of the funders for the present study and, if applicable, for the original study on which the present article is based | Funding |

*Give information separately for exposed and unexposed groups.

**Note:** An Explanation and Elaboration article discusses each checklist item and gives methodological background and published examples of transparent reporting. The STROBE checklist is best used in conjunction with this article (freely available on the Web sites of PLoS Medicine at http://www.plosmedicine.org/, Annals of Internal Medicine at http://www.annals.org/, and Epidemiology at http://www.epidem.com/). Information on the STROBE Initiative is available at http://www.strobe-statement.org.
